# (*E*)-Methyl 2-[(4-nitro­phen­yl)­hydrazono]­propanoate

**DOI:** 10.1107/S1600536807067268

**Published:** 2007-12-21

**Authors:** Hai-Yang Yu, Xin Fang, Ming-Lei Cao, Yan-Jun Zhang, Jun-Dong Wang

**Affiliations:** a Department of Chemistry, University of Fuzhou, Fuzhou 350002, People’s Republic of China

## Abstract

The title compound, C_10_H_11_N_3_O_4_, is a condensation product of 4-nitro­phenyl­hydrazine and methyl pyruvate. The complete mol­ecule except for the methyl groups can be considered as a conjugated π system. All non-H atoms are approximately coplanar (r.m.s. deviation 0.117 Å). The crystal packing involves an N—H⋯O hydrogen bond and a π–π inter­action between the aromatic rings, with a centroid–centroid distance of 3.617 Å.

## Related literature

For related literature, see: Humphrey & Kuethe (2006 ▶); Tietze *et al.* (2003 ▶); Van Order & Lindwall (1942 ▶).
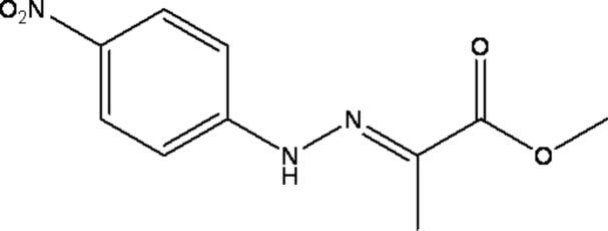


## Experimental

### 

#### Crystal data

C_10_H_11_N_3_O_4_
*M* *_r_* = 237.22
Monoclinic, 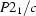

*a* = 12.836 (3) Å
*b* = 6.9260 (14) Å
*c* = 11.915 (2) Å
β = 90.11 (3)°
*V* = 1059.3 (4) Å^3^
*Z* = 4
Mo *K*α radiation
μ = 0.12 mm^−1^
*T* = 173 (2) K
0.60 × 0.54 × 0.16 mm

#### Data collection

Rigaku R-AXIS SPIDER diffractometer
Absorption correction: none
9730 measured reflections
2416 independent reflections
1997 reflections with *I* > 2σ(*I*)
*R*_int_ = 0.021

#### Refinement

*R*[*F* ^2^ > 2σ(*F* ^2^)] = 0.042
*wR*(*F* ^2^) = 0.126
*S* = 1.09
2416 reflections
176 parameters
H atoms treated by a mixture of independent and constrained refinement
Δρ_max_ = 0.29 e Å^−3^
Δρ_min_ = −0.29 e Å^−3^

### 

Data collection: *RAPID-AUTO* (Rigaku, 2004 ▶); cell refinement: *RAPID-AUTO*; data reduction: *RAPID-AUTO*; program(s) used to solve structure: *SHELXS97* (Sheldrick, 1990 ▶); program(s) used to refine structure: *SHELXL97* (Sheldrick, 1997 ▶); molecular graphics: *ORTEX* (McArdle, 1995 ▶); software used to prepare material for publication: *SHELXL97*.

## Supplementary Material

Crystal structure: contains datablocks I, global. DOI: 10.1107/S1600536807067268/bt2665sup1.cif

Structure factors: contains datablocks I. DOI: 10.1107/S1600536807067268/bt2665Isup2.hkl

Additional supplementary materials:  crystallographic information; 3D view; checkCIF report

## Figures and Tables

**Table 1:** Hydrogen-bond geometry (Å, °)

*D*—H⋯*A*	*D*—H	H⋯*A*	*D*⋯*A*	*D*—H⋯*A*
N2—H5⋯O3^i^	0.853 (18)	2.200 (18)	2.9928 (17)	154.6 (16)

Symmetry code: (i) 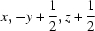.

**Fig. 1.**
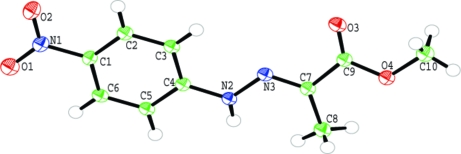
The molecular structure of the title compound with atom labels and 50% probability displacement ellipsoids for non-H atoms.

## supplementary crystallographic information

### Comment

The title compound, a phenylhydrazone derivative, is an important intermediate for the synthesis of indoles by the Fischer indole reaction (Van Order & Lindwall, 1942; Humphrey & Kuethe, 2006).

The molecular structure of the title compound is shown in Fig. 1. The complete molecule except the methyl groups can be considered as a conjugated π-system. All non-H atoms lie in a common plane (r.m.s. deviation 0.117 Å). The crystal packing shows an N—H···O hydrogen bond (Table 1) and a π-π interaction between the aromatic rings with a centroid-centroid distance of 3.617Å (symmetry operator: 1 - *x*, -*y*, 1 - *z*).

### Experimental

A suspension of 4-nitrophenylhydrazine (7.65 g, 50 mmol) in concd. HCl (20 ml) and H_2_O (20 ml) was heated to reflux untill the suspension solved. The solution was cooled to room temperature. Then the precipitate was filtrated off and dried. The solid was dissolved in methanol (100 ml) and treated with NaOAc (4.92 g, 60 mmol) and methyl pyruvate (5.10 g, 50 mmol). The mixture was stirred at room temperature for 18 h. Then the yellow precipitate was filtered off, washed with methanol and dried to afford 11.13 g of the title compound (47 mmol, 94%) (Tietze *et al.*, 2003). mp: 209.6–211.1°C. IR: (KBr, ν, cm^-1^): 3301 (N—H), 2962 (C—H), 1716 (C—O), 1611 (C—N), 1578, 1504, 1486, 1438, 1338, 1399, 1253, 1177, 1130, 1113, 847, 751.

### Refinement

H atoms of the two methyl groups were refined using a riding model with C—H = 0.96Å and U(H)=1.5*U*_eq_(C). These methyl groups were allowed to rotate but not to tip. All other H atoms were freely refined.

### Crystal data

**Table:**

C_10_H_11_N_3_O_4_	*F*_000_ = 496
*M**_r_* = 237.22	*D*_x_ = 1.487 Mg m^−^^3^
Monoclinic, *P*2_1_/*c*	Mo *K*α radiation λ = 0.71069 Å
Hall symbol: -P2ybc	Cell parameters from 7757 reflections
*a* = 12.836 (3) Å	θ = 6.4–55.0º
*b* = 6.9260 (14) Å	µ = 0.12 mm^−^^1^
*c* = 11.915 (2) Å	*T* = 173 (2) K
β = 90.11 (3)º	Chip, yellow
*V* = 1059.3 (4) Å^3^	0.60 × 0.54 × 0.16 mm
*Z* = 4	

### Data collection

**Table:**

Rigaku R-AXIS Spider diffractometer	2416 independent reflections
Radiation source: Rotating Anode	1997 reflections with *I* > 2σ(*I*)
Monochromator: graphite	*R*_int_ = 0.021
Detector resolution: 10 pixels mm^-1^	θ_max_ = 27.5º
*T* = 173(2) K	θ_min_ = 3.2º
ω oscillation scans	*h* = −16→16
Absorption correction: none	*k* = −8→7
9730 measured reflections	*l* = −15→15

### Refinement

**Table:**

Refinement on *F*^2^	Secondary atom site location: difference Fourier map
Least-squares matrix: full	Hydrogen site location: inferred from neighbouring sites
*R*[*F*^2^ > 2σ(*F*^2^)] = 0.042	H atoms treated by a mixture of independent and constrained refinement
*wR*(*F*^2^) = 0.126	*w* = 1/[σ^2^(*F*_o_^2^) + (0.0696*P*)^2^ + 0.336*P*] where *P* = (*F*_o_^2^ + 2*F*_c_^2^)/3
*S* = 1.09	(Δ/σ)_max_ < 0.001
2416 reflections	Δρ_max_ = 0.29 e Å^−^^3^
176 parameters	Δρ_min_ = −0.29 e Å^−^^3^
Primary atom site location: structure-invariant direct methods	Extinction correction: none

### Special details

**Table:**

Geometry. All e.s.d.'s (except the e.s.d. in the dihedral angle between two l.s. planes) are estimated using the full covariance matrix. The cell e.s.d.'s are taken into account individually in the estimation of e.s.d.'s in distances, angles and torsion angles; correlations between e.s.d.'s in cell parameters are only used when they are defined by crystal symmetry. An approximate (isotropic) treatment of cell e.s.d.'s is used for estimating e.s.d.'s involving l.s. planes.
Refinement. Refinement of *F*^2^ against ALL reflections. The weighted *R*-factor *wR* and goodness of fit *S* are based on *F*^2^, conventional *R*-factors *R* are based on *F*, with *F* set to zero for negative *F*^2^. The threshold expression of *F*^2^ > σ(*F*^2^) is used only for calculating *R*-factors(gt) *etc*. and is not relevant to the choice of reflections for refinement. *R*-factors based on *F*^2^ are statistically about twice as large as those based on *F*, and *R*- factors based on ALL data will be even larger.

### Fractional atomic coordinates and isotropic or equivalent isotropic displacement parameters (Å^2^)

**Table:**

	*x*	*y*	*z*	*U*_iso_*/*U*_eq_	
N1	0.23523 (9)	0.09076 (17)	0.62405 (10)	0.0249 (3)	
N2	0.65298 (8)	0.28388 (17)	0.55245 (9)	0.0217 (3)	
H5	0.6937 (13)	0.255 (3)	0.6065 (15)	0.028 (4)*	
N3	0.68657 (8)	0.33719 (16)	0.44935 (9)	0.0202 (3)	
O1	0.21019 (8)	0.03458 (18)	0.71835 (9)	0.0362 (3)	
O2	0.17278 (8)	0.10441 (19)	0.54620 (10)	0.0381 (3)	
O3	0.75514 (8)	0.43209 (18)	0.24033 (8)	0.0339 (3)	
O4	0.90897 (7)	0.50736 (15)	0.31757 (8)	0.0256 (3)	
C1	0.34331 (10)	0.14249 (18)	0.60429 (11)	0.0200 (3)	
C2	0.37287 (10)	0.2085 (2)	0.49903 (11)	0.0219 (3)	
H1	0.3235 (14)	0.217 (3)	0.4375 (16)	0.040 (5)*	
C3	0.47596 (10)	0.25744 (19)	0.48098 (11)	0.0207 (3)	
H2	0.4965 (13)	0.309 (2)	0.4086 (15)	0.029 (4)*	
C4	0.54879 (9)	0.23761 (19)	0.56748 (10)	0.0188 (3)	
C5	0.51738 (10)	0.1701 (2)	0.67302 (11)	0.0225 (3)	
H3	0.5685 (14)	0.158 (3)	0.7310 (16)	0.035 (5)*	
C6	0.41453 (10)	0.1227 (2)	0.69133 (11)	0.0225 (3)	
H4	0.3938 (13)	0.072 (3)	0.7635 (15)	0.031 (4)*	
C7	0.78391 (10)	0.37883 (19)	0.43797 (11)	0.0204 (3)	
C8	0.86529 (11)	0.3763 (3)	0.52797 (12)	0.0333 (4)	
H6	0.8767	0.5053	0.5548	0.050*	
H7	0.9291	0.3258	0.4980	0.050*	
H8	0.8423	0.2962	0.5888	0.050*	
C9	0.81181 (10)	0.44003 (19)	0.32127 (11)	0.0204 (3)	
C10	0.94452 (11)	0.5806 (2)	0.21009 (12)	0.0303 (3)	
H9	1.0130	0.6345	0.2185	0.045*	
H10	0.8975	0.6788	0.1841	0.045*	
H11	0.9466	0.4770	0.1566	0.045*	

### Atomic displacement parameters (Å^2^)

**Table:**

	*U*^11^	*U*^22^	*U*^33^	*U*^12^	*U*^13^	*U*^23^
N1	0.0205 (6)	0.0267 (6)	0.0276 (6)	−0.0007 (5)	0.0051 (4)	−0.0034 (5)
N2	0.0181 (5)	0.0302 (6)	0.0169 (5)	−0.0020 (5)	0.0008 (4)	0.0016 (4)
N3	0.0202 (5)	0.0217 (6)	0.0188 (5)	−0.0005 (4)	0.0040 (4)	−0.0011 (4)
O1	0.0273 (5)	0.0512 (7)	0.0302 (6)	−0.0087 (5)	0.0102 (4)	0.0038 (5)
O2	0.0202 (5)	0.0564 (8)	0.0376 (6)	−0.0041 (5)	−0.0033 (4)	0.0014 (5)
O3	0.0255 (5)	0.0559 (7)	0.0202 (5)	−0.0093 (5)	−0.0011 (4)	0.0031 (5)
O4	0.0199 (5)	0.0350 (6)	0.0220 (5)	−0.0066 (4)	0.0039 (3)	0.0013 (4)
C1	0.0174 (6)	0.0199 (6)	0.0227 (6)	−0.0011 (5)	0.0036 (5)	−0.0039 (5)
C2	0.0204 (6)	0.0246 (7)	0.0208 (6)	0.0013 (5)	−0.0005 (5)	−0.0005 (5)
C3	0.0216 (6)	0.0234 (6)	0.0172 (6)	0.0006 (5)	0.0026 (5)	0.0017 (5)
C4	0.0186 (6)	0.0184 (6)	0.0193 (6)	0.0000 (5)	0.0031 (4)	−0.0022 (5)
C5	0.0210 (6)	0.0293 (7)	0.0173 (6)	−0.0004 (5)	0.0001 (5)	−0.0010 (5)
C6	0.0235 (6)	0.0266 (7)	0.0173 (6)	−0.0014 (5)	0.0045 (5)	−0.0006 (5)
C7	0.0195 (6)	0.0217 (6)	0.0199 (6)	−0.0017 (5)	0.0019 (5)	−0.0024 (5)
C8	0.0234 (6)	0.0543 (10)	0.0223 (7)	−0.0100 (7)	−0.0005 (5)	0.0040 (6)
C9	0.0191 (6)	0.0216 (6)	0.0207 (6)	−0.0007 (5)	0.0028 (5)	−0.0022 (5)
C10	0.0256 (7)	0.0384 (8)	0.0270 (7)	−0.0052 (6)	0.0085 (5)	0.0057 (6)

### Geometric parameters (Å, °)

**Table:**

N1—O2	1.2284 (17)	C3—C4	1.3970 (18)
N1—O1	1.2323 (16)	C3—H2	0.970 (18)
N1—C1	1.4525 (16)	C4—C5	1.4016 (18)
N2—N3	1.3540 (15)	C5—C6	1.3783 (18)
N2—C4	1.3872 (16)	C5—H3	0.956 (18)
N2—H5	0.853 (18)	C6—H4	0.966 (18)
N3—C7	1.2897 (16)	C7—C8	1.4958 (19)
O3—C9	1.2080 (17)	C7—C9	1.4977 (18)
O4—C9	1.3323 (15)	C8—H6	0.9600
O4—C10	1.4518 (16)	C8—H7	0.9600
C1—C6	1.3880 (19)	C8—H8	0.9600
C1—C2	1.3885 (19)	C10—H9	0.9600
C2—C3	1.3831 (17)	C10—H10	0.9600
C2—H1	0.970 (19)	C10—H11	0.9600
			
O2—N1—O1	122.81 (12)	C4—C5—H3	118.7 (11)
O2—N1—C1	118.73 (11)	C5—C6—C1	119.20 (12)
O1—N1—C1	118.46 (12)	C5—C6—H4	119.5 (10)
N3—N2—C4	119.27 (11)	C1—C6—H4	121.3 (10)
N3—N2—H5	123.6 (11)	N3—C7—C8	126.68 (12)
C4—N2—H5	116.0 (11)	N3—C7—C9	113.23 (11)
C7—N3—N2	117.81 (11)	C8—C7—C9	120.07 (11)
C9—O4—C10	116.57 (11)	C7—C8—H6	109.5
C6—C1—C2	121.79 (12)	C7—C8—H7	109.5
C6—C1—N1	118.84 (12)	H6—C8—H7	109.5
C2—C1—N1	119.37 (12)	C7—C8—H8	109.5
C3—C2—C1	118.97 (12)	H6—C8—H8	109.5
C3—C2—H1	119.4 (11)	H7—C8—H8	109.5
C1—C2—H1	121.6 (11)	O3—C9—O4	123.49 (12)
C2—C3—C4	120.02 (12)	O3—C9—C7	125.70 (12)
C2—C3—H2	119.3 (10)	O4—C9—C7	110.81 (11)
C4—C3—H2	120.6 (10)	O4—C10—H9	109.5
N2—C4—C3	121.74 (12)	O4—C10—H10	109.5
N2—C4—C5	118.16 (12)	H9—C10—H10	109.5
C3—C4—C5	120.10 (12)	O4—C10—H11	109.5
C6—C5—C4	119.92 (12)	H9—C10—H11	109.5
C6—C5—H3	121.4 (11)	H10—C10—H11	109.5

### Hydrogen-bond geometry (Å, °)

**Table:**

*D*—H···*A*	*D*—H	H···*A*	*D*···*A*	*D*—H···*A*
N2—H5···O3^i^	0.853 (18)	2.200 (18)	2.9928 (17)	154.6 (16)

Symmetry codes: (i) *x*, −*y*+1/2, *z*+1/2.
